# Literary Notes

**Published:** 1896-01

**Authors:** 


### Literary Notes.

E. B. Treat, Publisher, New York, has in press for early publication the 1896 *International Medical Annual*, being the fourteenth yearly issue of this handy and useful work. Since the first issue of this one volume reference work, each year has witnessed marked improvements ; and the prospectus of the forthcoming volume gives promise that it will surpass any of its predecessors. It will be the conjoint authorship of forty prominent specialists, selected from among the eminent physicians and surgeons of America, England and the Continent. It will contain reports of the progress of medical science at home and abroad, together with a large number of original articles and reviews on subjects with which the several authors are especially associated. In short, the design of the book is, while not neglecting the specialist, to bring the general practitioner into direct communication with those who are advancing the science of medicine, so he may be furnished with all that is worthy of preservation, as reliable aids in his daily work. Illustrations in black and colors will be used wherever helpful in elucidating the text. Altogether it makes a most useful, if not absolutely indispensable, investment for the physician. The price will remain the same as previous issues, $2.75.

---

Keil's Medical Pharmaceutical and Dental Directory.—George Keil, editor, Philadelphia, announces the early publication (fourth edition) of Keil's Medical, Pharmaceutical and Dental Register-Directory and Intelligencer, for Pennsylvania, New York, New Jersey, Maryland, Delaware and District of Columbia. Its list of national colleges, state hospitals, homes, dispensaries, societies, and post-office addresses of physicians, druggists and dentists, school of graduation and year, all the latest laws in these states, will be complete to date of issue as a personal canvass will be made for data. The names in large cities, in addition to being in alphabetical order, will be numerically arranged by streets, also an alphabetical list of names of the whole directory, giving the page of each, features that will no doubt be appreciated.

---

The *American Medical Review*, edited by Dr. Daniel Lewis, president of the New York state board of health, and published by the R. N. Plummer Company, 106–108 Fulton street, New York, has issued its first number bearing date January, 1896. It is a beautiful specimen of magazine printing and its contents bear the stamp of experienced editorial management. It is occupying a unique field in medical literature similar to that of the *Review of Reviews*, in its relation to general literature. If this medical review of reviews keeps itself within its original limitations, it is certain to meet with the favor of the medical profession.

---

What is generally conceded in Philadelphia to be one of the most desirable building sites in the city has lately been purchased by

*The Ladies' Home Journal.* The property is located at Sixth and Walnut streets, which means that it fronts on two of the most beautiful squares in Philadelphia, the famous Independence Square on the east and Washington Square on the south. The land acquired includes five properties. On May 1, 1896, the houses thereon will be torn down to make room for a building costing $250,000, to be solely owned and exclusively occupied by the *Journal.* The building will require two years in its construction.

---

The *Quarterly Medical Journal* for Yorkshire and adjoining counties has lately come to our table as an exchange. It is edited for the promoters by Mr. Simeon Snell, who has an able corps of assistants and associates. It is published at Sheffield, Eng., by Pawson and Brailsford. It is a handsome magazine, ably edited and well printed. We are glad to welcome it to our exchange table.

---

Bulletin No. 8 of the Harvard Medical Alumni Association contains a report of the fifth annual meeting, held at Boston, June 25, 1895. At the annual dinner, speeches were made by the president, Dr. George B. Shattuck, President Elliott, Dr. Roswell Park, of Buffalo, and others.

---

The *American Journal of Surgery and Gynecology* has been removed to St. Louis. Dr. Emory Lanphear, professor of surgery in the Woman's Medical College, has been appointed editor-in-chief.

---

The *College and Clinical Record* will be hereafter known under the name of *Dunglison's College and Clinical Record:* a Monthly Journal of Practical Medicine.

---

The *Medical News*, for more than thirty years published in Philadelphia by Henry C. Lea and his successors, Lea Brothers & Company, announces that it will remove its editorial and publication offices to New York City with the beginning of the New Year.

